# Crystal structure of *N*′-[2-(benzo[*d*]thia­zol-2-yl)acet­yl]-4-methyl­benzene­sulfono­hydrazide

**DOI:** 10.1107/S2056989017008738

**Published:** 2017-06-16

**Authors:** Rasha A. Azzam, Galal H. Elgemeie, Rasha E. Elsayed, Peter G. Jones

**Affiliations:** a Chemistry Department, Faculty of Science, Helwan University, Cairo, Egypt; b Institut für Anorganische und Analytische Chemie, Technische Universität Braunschweig, Postfach 3329, D-38023 Braunschweig, Germany

**Keywords:** crystal structure, benzo­thia­zole, hydrazide

## Abstract

In the title compound, C_16_H_15_N_3_O_3_S_2_, the hydrazide N atom bonded to the C=O group is planar, whereas that bonded to the SO_2_ group is pyramidally coordinated. The inter­planar angle between the ring systems is 40.71 (3)°. Mol­ecules are connected into ribbons parallel to the *b* axis by two classical hydrogen bonds N—H⋯O=C and N—H⋯N_thia­zole_.

## Abstract 2

In the title compound, the hydrazide N atom bonded to the C=O group is planar, whereas that bonded to the SO_2_ group is pyramidally coordinated. The inter­planar angle between the ring systems is 40.71 (3)°. In the crystal, mol­ecules are connected by N—H⋯O=C and N—H⋯N_thia­zole_ hydrogen bonds, forming ribbons parallel to the *b* axis.

## Chemical context   

Benzo­thia­zoles are versatile heterocyclic compounds with potential pharmaceutical applications (Elgemeie & Aal, 1986 ▸). Various benzo­thia­zoles have been used as anti-inflammatory, anti­microbial and analgesic agents and as laser dyes (Elgemeie, 1989 ▸). This has led to an increasing inter­est in benzo­thia­zole derivatives in the area of drug design and discovery (Elgemeie *et al.*, 2000 ▸). As a part of our research work on new syntheses of benzo­thia­zoles as chemotherapeutic agents (Elgemeie *et al.*, 2017 ▸), we have previously reported the synthesis of 2-aryl­benzo­thia­zoles that later found applications as anti­cancer agents and are presently in clinical use for various diseases (Elgemeie & Elghandour, 1990 ▸). We report here the new compound *N*′-(2-(benzo[*d*]thia­zol-2-yl)acet­yl)-4-methyl­benzene­sulfono­hydrazide (**1**), which was prepared by the reaction of 2-(benzo[*d*]thia­zol-2-yl)acetohydrazide with *p*-toluene­sulfonyl chloride in the presence of pyridine at room temperature. The structure of (**1**) was determined on the basis of its spectroscopic data and elemental analysis (see *Experimental*). In order to establish the structure of the product unambiguously, its crystal structure was determined.
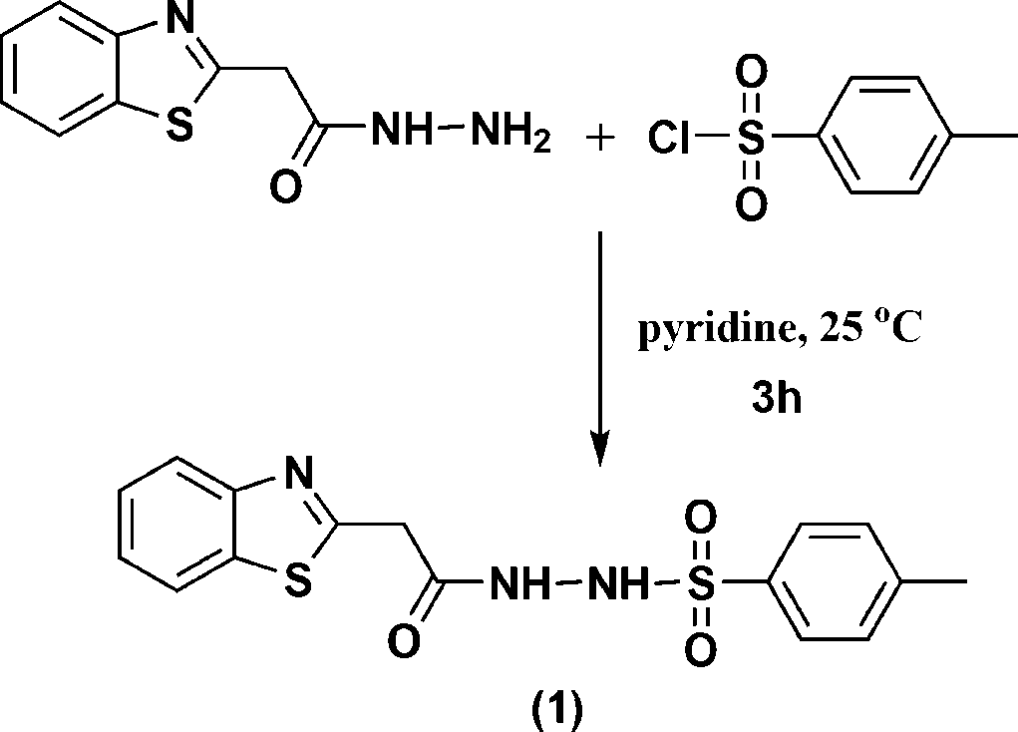


## Structural commentary   

**Figure 1:**
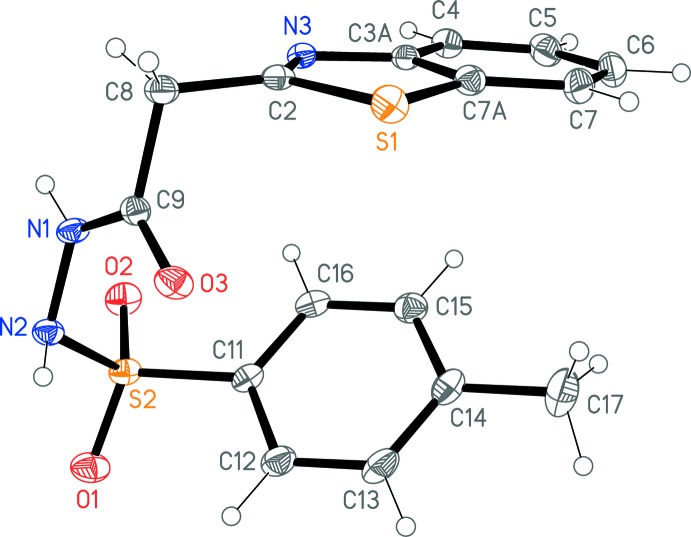
The structure of compound (**1**) in the crystal, with displacement ellipsoids at the 50% probability level.

The X-ray analysis confirms the exclusive presence of the form (**1**) in the solid state (Fig. 1 ▸). The mol­ecular dimensions may be regarded as normal (Table 1 ▸); the torsion angles defining the conformation of the chain connecting the ring systems are also given in this Table. The bond lengths C2—S1 and C2—N3 in the heterocycle correspond well with the average values of 1.750 (15) and 1.200 (14) Å found in the Cambridge Structural Database (Version 5.38; Groom *et al.*, 2016 ▸) for 375 examples of this ring system (unsubstituted benzo ring, carbon-substituted at C2). Nitro­gen N1 displays a planar geometry, whereas N2 is pyramidal [they lie 0.014 (7) and 0.337 (8) Å, respectively, outside the plane of their substituents]. Hydrogen atom H01 is anti­periplanar to O3 and H02 to O2 across the N1—C9 and N2—S2 bonds, respectively. The inter­planar angle between the ring systems is 40.71 (3)°.

## Supra­molecular features   

**Figure 2:**
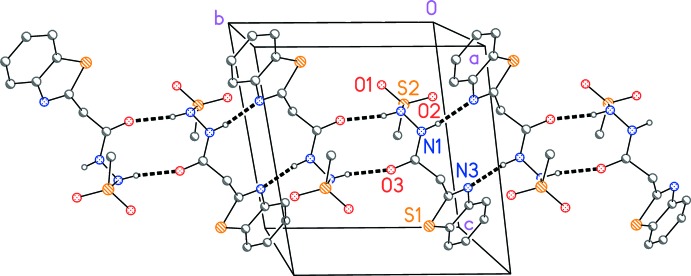
Packing diagram of compound (**1**), viewed perpendicular to the *bc* plane. Hydrogen bonds are drawn as thick dashed lines. H atoms not involved in hydrogen bonds have been omitted for clarity.

Mol­ecules are connected by two pairs of classical hydrogen bonds across inversion centres, to form ribbons parallel to the *b* axis (Table 2 ▸, Fig. 2 ▸). A C—H⋯O inter­action connects the mol­ecules by *c*-axis translation (not shown in the Figure), forming layers parallel to (100).

## Database survey   

A search of the Cambridge Database (Version 5.38; Groom *et al.*, 2016 ▸) for the substructure Ar—SO_2_—NH—NH—C(=O)—C gave six hits: EYOZIB, KUKYOG, XOVFEV, XOZDOG, YOTKAU and ZIVVUX.

## Synthesis and crystallization   

A solution of *p*-toluene­sulfonyl chloride (1.90 g, 0.015 mol) in pyridine (10 ml) was added gradually to a stirred solution of 2-(benzo[*d*]thia­zol-2-yl)acetohydrazide (2.07 g, 0.01 mol) in pyridine (10 ml) at 273 K. The reaction mixture was then stirred at room temperature for 3 h (TLC control). After the reaction was completed, the mixture was poured into ice-water with continuous stirring and neutralized with 1 *N* HCl solution to pH 7. The precipitate thus formed was filtered off, washed with water and recrystallized from ethanol to give colourless crystals (yield 85%; m.p. = 458 K). IR (KBr, cm^−1^): ν 3427 (NH), 3164 (Ar CH), 2929, 2858 (CH_3_, CH_2_), 1692 (C=O); ^1^H NMR (400 MHz, DMSO-*d*_6_): *δ* 2.26 (*s*, 3H, CH_3_), 3.95 (*s*, 2H, CH_2_), 7.15 (*d*, *J* = 8 Hz, 2H, SO_2_C_6_H_4_), 7.44 (*t*, *J* = 8 Hz, 1H, benzo­thia­zole H), 7.52 (*t*, *J* = 8 Hz, 1H, benzo­thia­zole H), 7.62 (*d*, *J* = 8 Hz, 2H, SO_2_C_6_H_4_), 7.96 (*d*, *J* = 8 Hz, 1H, benzo­thia­zole H), 8.07 (*d*, *J* = 8 Hz, 1H, benzo­thia­zole H), 9.95 (*s*, 1H, NH), 10.52 (*s*, 1H, NH).

## Refinement   

Crystal data, data collection and structure refinement details are summarized in Table 3 ▸. NH hydrogen atoms were refined freely. The methyl hydrogen atoms were not well defined and so were refined as a hexa­gon of half-occupied sites with C—H = 0.98 Å (AFIX 127). Other hydrogen atoms were included using a riding model starting from calculated positions (C—H_aromatic_ = 0.95, C—H_methyl­ene_ 0.99 Å) with *U*_iso_(H) = 1.2*U*_eq_(C).

Despite the slightly larger ellipsoid of the benzo­thia­zol sulfur atom S1, there is no evidence for significant mixing (disorder) of the sites N3/S1.

## Supplementary Material

Crystal structure: contains datablock(s) I. DOI: 10.1107/S2056989017008738/hg5489sup1.cif

Structure factors: contains datablock(s) I. DOI: 10.1107/S2056989017008738/hg5489Isup2.hkl

Supporting information file. DOI: 10.1107/S2056989017008738/hg5489Isup3.cml

CCDC reference: 1555516

Additional supporting information:  crystallographic information; 3D view; checkCIF report

## Figures and Tables

**Table 1:** Selected geometric parameters (Å, °)

S1—C2	1.7373 (11)	N1—N2	1.4069 (12)
C2—N3	1.2972 (14)		
			
C7*A*—S1—C2	89.39 (5)	N1—N2—S2	112.94 (7)
C9—N1—N2	121.14 (9)		
			
S1—C2—C8—C9	−80.57 (10)	N2—S2—C11—C12	77.16 (9)
C2—C8—C9—N1	−109.79 (10)	H01—N1—N2—H02	−146.7 (17)
C8—C9—N1—N2	176.74 (9)	O3—C9—N1—H01	175.5 (13)
C9—N1—N2—S2	−96.08 (10)	H02—N2—S2—O2	179.1 (12)
N1—N2—S2—C11	62.53 (8)		

**Table 2:** Hydrogen-bond geometry (Å, °)

*D*—H⋯*A*	*D*—H	H⋯*A*	*D*⋯*A*	*D*—H⋯*A*
N1—H01⋯N3^i^	0.866 (16)	2.013 (16)	2.8717 (13)	171.0 (15)
N2—H02⋯O3^ii^	0.845 (17)	2.029 (17)	2.8553 (12)	165.7 (16)
C6—H6⋯O2^iii^	0.95	2.54	3.4142 (15)	154

Symmetry codes: (i) 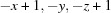; (ii) 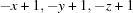; (iii) 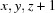.

**Table 3:** Experimental details

Crystal data	
Chemical formula	C_16_H_15_N_3_O_3_S_2_
*M*_r_	361.43
Crystal system, space group	Triclinic, *P* 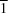
Temperature (K)	100
*a*, *b*, *c* (Å)	8.3436 (4), 9.7591 (5), 10.8815 (6)
α, β, γ (°)	97.905 (4), 98.142 (4), 101.576 (4)
*V* (Å^3^)	846.59 (8)
*Z*	2
Radiation type	Mo *K*α
μ (mm^−1^)	0.33
Crystal size (mm)	0.5 × 0.4 × 0.2

Data collection	
Diffractometer	Oxford Diffraction Xcalibur Eos
Absorption correction	Multi-scan (*CrysAlis PRO*; Rigaku Oxford Diffraction, 2015 ▸)
*T*_min_, *T*_max_	0.952, 1.000
No. of measured, independent and observed [*I* > 2σ(*I*)] reflections	45592, 5040, 4503
*R*_int_	0.032
(sin θ/λ)_max_ (Å^−1^)	0.726

Refinement	
*R*[*F* ^2^ > 2σ(*F* ^2^)], *wR*(*F* ^2^), *S*	0.031, 0.079, 1.05
No. of reflections	5040
No. of parameters	226
H-atom treatment	H atoms treated by a mixture of independent and constrained refinement
Δρ_max_, Δρ_min_ (e Å^−3^)	0.41, −0.42

Computer programs: *CrysAlis PRO* (Rigaku OD, 2015 ▸), *SHELXS97* and *SHELXL97* (Sheldrick, 2008 ▸) and *XP* (Siemens, 1994 ▸).

## supplementary crystallographic information

### Crystal data

**Table:**

C_16_H_15_N_3_O_3_S_2_	*Z* = 2
*M**_r_* = 361.43	*F*(000) = 376
Triclinic, *P*1	*D*_x_ = 1.418 Mg m^−^^3^
Hall symbol: -P 1	Mo *K*α radiation, λ = 0.71073 Å
*a* = 8.3436 (4) Å	Cell parameters from 13640 reflections
*b* = 9.7591 (5) Å	θ = 2.6–30.8°
*c* = 10.8815 (6) Å	µ = 0.33 mm^−^^1^
α = 97.905 (4)°	*T* = 100 K
β = 98.142 (4)°	Tablet, colourless
γ = 101.576 (4)°	0.5 × 0.4 × 0.2 mm
*V* = 846.59 (8) Å^3^	

### Data collection

**Table:**

Oxford Diffraction Xcalibur Eos diffractometer	5040 independent reflections
Radiation source: fine-focus sealed X-ray tube	4503 reflections with *I* > 2σ(*I*)
Graphite monochromator	*R*_int_ = 0.032
Detector resolution: 16.1419 pixels mm^-1^	θ_max_ = 31.1°, θ_min_ = 2.5°
ω–scan	*h* = −12→11
Absorption correction: multi-scan (CrysAlis PRO; Rigaku Oxford Diffraction, 2015)	*k* = −14→14
*T*_min_ = 0.952, *T*_max_ = 1.000	*l* = −15→15
45592 measured reflections	

### Refinement

**Table:**

Refinement on *F*^2^	Primary atom site location: structure-invariant direct methods
Least-squares matrix: full	Secondary atom site location: difference Fourier map
*R*[*F*^2^ > 2σ(*F*^2^)] = 0.031	Hydrogen site location: inferred from neighbouring sites
*wR*(*F*^2^) = 0.079	H atoms treated by a mixture of independent and constrained refinement
*S* = 1.05	*w* = 1/[σ^2^(*F*_o_^2^) + (0.0354*P*)^2^ + 0.355*P*] where *P* = (*F*_o_^2^ + 2*F*_c_^2^)/3
5040 reflections	(Δ/σ)_max_ = 0.008
226 parameters	Δρ_max_ = 0.41 e Å^−^^3^
0 restraints	Δρ_min_ = −0.42 e Å^−^^3^

### Special details

**Table:**

Geometry. All esds (except the esd in the dihedral angle between two l.s. planes) are estimated using the full covariance matrix. The cell esds are taken into account individually in the estimation of esds in distances, angles and torsion angles; correlations between esds in cell parameters are only used when they are defined by crystal symmetry. An approximate (isotropic) treatment of cell esds is used for estimating esds involving l.s. planes. Least-squares planes (x,y,z in crystal coordinates) and deviations from them (* indicates atom used to define plane) 6.2088 (0.0012) x - 6.2111 (0.0022) y + 4.5043 (0.0028) z = 5.8783 (0.0023) * -0.0257 (0.0005) S1 * -0.0152 (0.0007) C2 * 0.0114 (0.0007) N3 * 0.0250 (0.0009) C3A * -0.0026 (0.0008) C4 * -0.0272 (0.0009) C5 * -0.0057 (0.0009) C6 * 0.0176 (0.0009) C7 * 0.0223 (0.0009) C7A Rms deviation of fitted atoms = 0.0190 7.8014 (0.0014) x - 0.3099 (0.0046) y + 1.6293 (0.0049) z = 2.0216 (0.0030) Angle to previous plane (with approximate esd) = 40.71 ( 0.03 ) * 0.0051 (0.0008) C11 * -0.0053 (0.0008) C12 * -0.0012 (0.0008) C13 * 0.0080 (0.0008) C14 * -0.0083 (0.0008) C15 * 0.0018 (0.0008) C16 Rms deviation of fitted atoms = 0.0057=========================================================== Least-squares planes (x,y,z in crystal coordinates) and deviations from them (* indicates atom used to define plane) 6.5691 (0.0357) x + 3.5739 (0.0897) y - 5.4501 (0.0327) z = 1.7544 (0.0236) * 0.0000 (0.0001) C9 * 0.0000 (0.0000) H01 * 0.0000 (0.0000) N2 -0.0139 (0.0071) N1 Rms deviation of fitted atoms = 0.0000 - 2.5491 (0.0143) x + 2.7622 (0.0942) y + 9.8695 (0.0419) z = 3.6614 (0.0102) Angle to previous plane (with approximate esd) = 66.25 ( 0.42 ) * 0.0000 (0.0000) S2 * 0.0000 (0.0000) H02 * 0.0000 (0.0000) N1 -0.3372 (0.0082) N2 Rms deviation of fitted atoms = 0.0000
Refinement. Refinement of F^2^ against ALL reflections. The weighted R-factor wR and goodness of fit S are based on F^2^, conventional R-factors R are based on F, with F set to zero for negative F^2^. The threshold expression of F^2^ > 2sigma(F^2^) is used only for calculating R-factors(gt) etc. and is not relevant to the choice of reflections for refinement. R-factors based on F^2^ are statistically about twice as large as those based on F, and R- factors based on ALL data will be even larger.

### Fractional atomic coordinates and isotropic or equivalent isotropic displacement parameters (Å^2^)

**Table:**

	*x*	*y*	*z*	*U*_iso_*/*U*_eq_	Occ. (<1)
S1	0.57619 (4)	0.26397 (3)	0.86910 (3)	0.01947 (7)	
C2	0.56817 (13)	0.15226 (11)	0.72845 (10)	0.01492 (19)	
N3	0.46391 (11)	0.03031 (9)	0.70990 (9)	0.01448 (17)	
C3A	0.38014 (13)	0.01774 (11)	0.81106 (10)	0.01466 (19)	
C4	0.25456 (14)	−0.09823 (12)	0.81811 (11)	0.0185 (2)	
H4	0.2221	−0.1782	0.7522	0.022*	
C5	0.17867 (14)	−0.09401 (13)	0.92308 (12)	0.0223 (2)	
H5	0.0920	−0.1714	0.9287	0.027*	
C6	0.22767 (15)	0.02293 (14)	1.02157 (11)	0.0238 (2)	
H6	0.1749	0.0225	1.0934	0.029*	
C7	0.35125 (15)	0.13868 (13)	1.01602 (11)	0.0223 (2)	
H7	0.3840	0.2178	1.0827	0.027*	
C7A	0.42644 (13)	0.13542 (12)	0.90892 (10)	0.0170 (2)	
C8	0.66708 (13)	0.20010 (12)	0.63115 (11)	0.0172 (2)	
H8A	0.6814	0.1169	0.5740	0.021*	
H8B	0.7784	0.2573	0.6723	0.021*	
C9	0.57197 (12)	0.28914 (11)	0.55711 (10)	0.01401 (19)	
N1	0.50433 (11)	0.22734 (9)	0.43761 (9)	0.01544 (17)	
H01	0.519 (2)	0.1475 (18)	0.4007 (15)	0.027 (4)*	
N2	0.40574 (11)	0.29335 (10)	0.35950 (9)	0.01529 (17)	
H02	0.434 (2)	0.3829 (18)	0.3759 (15)	0.029 (4)*	
O1	0.12667 (11)	0.31786 (9)	0.27597 (8)	0.02335 (18)	
S2	0.20281 (3)	0.23646 (3)	0.35718 (2)	0.01622 (7)	
O2	0.16863 (11)	0.08446 (8)	0.32664 (8)	0.02168 (17)	
O3	0.55748 (10)	0.40588 (8)	0.60522 (8)	0.01870 (16)	
C11	0.16430 (13)	0.28095 (11)	0.51069 (11)	0.0163 (2)	
C12	0.15942 (14)	0.42100 (12)	0.55429 (12)	0.0203 (2)	
H12	0.1721	0.4901	0.5007	0.024*	
C13	0.13587 (15)	0.45785 (12)	0.67661 (12)	0.0233 (2)	
H13	0.1329	0.5532	0.7069	0.028*	
C14	0.11639 (14)	0.35757 (13)	0.75641 (11)	0.0208 (2)	
C15	0.11840 (13)	0.21746 (12)	0.71012 (11)	0.0196 (2)	
H15	0.1023	0.1477	0.7628	0.023*	
C16	0.14367 (13)	0.17876 (11)	0.58797 (11)	0.0176 (2)	
H16	0.1468	0.0835	0.5575	0.021*	
C17	0.09014 (19)	0.39948 (16)	0.88922 (13)	0.0327 (3)	
H17A	0.0428	0.4838	0.8946	0.049*	0.50
H17B	0.1969	0.4207	0.9468	0.049*	0.50
H17C	0.0137	0.3212	0.9127	0.049*	0.50
H17D	0.1261	0.3334	0.9415	0.049*	0.50
H17E	−0.0280	0.3964	0.8893	0.049*	0.50
H17F	0.1552	0.4960	0.9233	0.049*	0.50

### Atomic displacement parameters (Å^2^)

**Table:**

	*U*^11^	*U*^22^	*U*^33^	*U*^12^	*U*^13^	*U*^23^
S1	0.02314 (14)	0.01447 (12)	0.01758 (14)	0.00166 (10)	0.00006 (10)	−0.00091 (9)
C2	0.0156 (4)	0.0138 (4)	0.0160 (5)	0.0062 (4)	0.0003 (4)	0.0028 (4)
N3	0.0162 (4)	0.0134 (4)	0.0152 (4)	0.0061 (3)	0.0028 (3)	0.0029 (3)
C3A	0.0154 (4)	0.0149 (4)	0.0144 (5)	0.0061 (4)	0.0009 (4)	0.0027 (4)
C4	0.0177 (5)	0.0180 (5)	0.0193 (5)	0.0038 (4)	0.0019 (4)	0.0039 (4)
C5	0.0174 (5)	0.0285 (6)	0.0225 (6)	0.0043 (4)	0.0042 (4)	0.0101 (5)
C6	0.0215 (5)	0.0366 (7)	0.0168 (5)	0.0109 (5)	0.0054 (4)	0.0074 (5)
C7	0.0249 (6)	0.0279 (6)	0.0144 (5)	0.0104 (5)	0.0017 (4)	0.0003 (4)
C7A	0.0178 (5)	0.0173 (5)	0.0155 (5)	0.0053 (4)	0.0003 (4)	0.0016 (4)
C8	0.0150 (5)	0.0175 (5)	0.0207 (5)	0.0054 (4)	0.0028 (4)	0.0064 (4)
C9	0.0130 (4)	0.0125 (4)	0.0169 (5)	0.0018 (3)	0.0033 (4)	0.0043 (4)
N1	0.0184 (4)	0.0119 (4)	0.0170 (4)	0.0071 (3)	0.0025 (3)	0.0012 (3)
N2	0.0186 (4)	0.0122 (4)	0.0155 (4)	0.0053 (3)	0.0018 (3)	0.0020 (3)
O1	0.0244 (4)	0.0237 (4)	0.0210 (4)	0.0090 (3)	−0.0038 (3)	0.0031 (3)
S2	0.01722 (12)	0.01375 (12)	0.01595 (13)	0.00428 (9)	−0.00088 (9)	−0.00068 (9)
O2	0.0247 (4)	0.0139 (4)	0.0223 (4)	0.0018 (3)	0.0004 (3)	−0.0036 (3)
O3	0.0264 (4)	0.0110 (3)	0.0175 (4)	0.0041 (3)	0.0012 (3)	0.0010 (3)
C11	0.0134 (4)	0.0156 (5)	0.0194 (5)	0.0047 (4)	0.0020 (4)	−0.0002 (4)
C12	0.0219 (5)	0.0151 (5)	0.0258 (6)	0.0065 (4)	0.0074 (4)	0.0030 (4)
C13	0.0247 (6)	0.0163 (5)	0.0298 (6)	0.0070 (4)	0.0101 (5)	−0.0014 (4)
C14	0.0162 (5)	0.0230 (5)	0.0235 (6)	0.0056 (4)	0.0060 (4)	−0.0001 (4)
C15	0.0159 (5)	0.0206 (5)	0.0229 (6)	0.0053 (4)	0.0031 (4)	0.0049 (4)
C16	0.0149 (5)	0.0142 (5)	0.0228 (5)	0.0046 (4)	0.0006 (4)	0.0007 (4)
C17	0.0379 (7)	0.0349 (7)	0.0264 (7)	0.0088 (6)	0.0143 (6)	−0.0010 (5)

### Geometric parameters (Å, º)

**Table:**

S1—C7A	1.7310 (12)	C13—C14	1.3957 (18)
S1—C2	1.7373 (11)	C14—C15	1.3949 (16)
C2—N3	1.2972 (14)	C14—C17	1.5056 (17)
C2—C8	1.4996 (15)	C15—C16	1.3882 (16)
N3—C3A	1.3914 (14)	C4—H4	0.9500
C3A—C4	1.3983 (15)	C5—H5	0.9500
C3A—C7A	1.4040 (15)	C6—H6	0.9500
C4—C5	1.3819 (16)	C7—H7	0.9500
C5—C6	1.4037 (18)	C8—H8A	0.9900
C6—C7	1.3826 (18)	C8—H8B	0.9900
C7—C7A	1.3992 (16)	N1—H01	0.866 (16)
C8—C9	1.5238 (14)	N2—H02	0.845 (17)
C9—O3	1.2231 (13)	C12—H12	0.9500
C9—N1	1.3464 (14)	C13—H13	0.9500
N1—N2	1.4069 (12)	C15—H15	0.9500
N2—S2	1.6680 (10)	C16—H16	0.9500
O1—S2	1.4325 (8)	C17—H17A	0.9800
S2—O2	1.4353 (8)	C17—H17B	0.9800
S2—C11	1.7580 (11)	C17—H17C	0.9800
C11—C16	1.3907 (16)	C17—H17D	0.9800
C11—C12	1.3944 (15)	C17—H17E	0.9800
C12—C13	1.3821 (17)	C17—H17F	0.9800
			
C7A—S1—C2	89.39 (5)	C5—C6—H6	119.4
N3—C2—C8	122.61 (10)	C6—C7—H7	121.1
N3—C2—S1	115.98 (8)	C7A—C7—H7	121.1
C8—C2—S1	121.25 (8)	C2—C8—H8A	110.2
C2—N3—C3A	110.63 (9)	C9—C8—H8A	110.2
N3—C3A—C4	124.96 (10)	C2—C8—H8B	110.2
N3—C3A—C7A	114.86 (10)	C9—C8—H8B	110.2
C4—C3A—C7A	120.15 (10)	H8A—C8—H8B	108.5
C5—C4—C3A	118.54 (11)	C9—N1—H01	124.9 (11)
C4—C5—C6	120.98 (11)	N2—N1—H01	113.9 (11)
C7—C6—C5	121.26 (11)	N1—N2—H02	113.1 (11)
C6—C7—C7A	117.79 (11)	S2—N2—H02	111.0 (11)
C7—C7A—C3A	121.26 (11)	C13—C12—H12	120.5
C7—C7A—S1	129.57 (9)	C11—C12—H12	120.5
C3A—C7A—S1	109.14 (8)	C12—C13—H13	119.4
C2—C8—C9	107.51 (8)	C14—C13—H13	119.4
O3—C9—N1	123.90 (10)	C16—C15—H15	119.6
O3—C9—C8	121.56 (10)	C14—C15—H15	119.6
N1—C9—C8	114.53 (9)	C15—C16—H16	120.4
C9—N1—N2	121.14 (9)	C11—C16—H16	120.4
N1—N2—S2	112.94 (7)	C14—C17—H17A	109.5
O1—S2—O2	120.92 (5)	C14—C17—H17B	109.5
O1—S2—N2	103.98 (5)	H17A—C17—H17B	109.5
O2—S2—N2	106.20 (5)	C14—C17—H17C	109.5
O1—S2—C11	109.49 (5)	H17A—C17—H17C	109.5
O2—S2—C11	107.66 (5)	H17B—C17—H17C	109.5
N2—S2—C11	107.89 (5)	C14—C17—H17D	109.5
C16—C11—C12	120.86 (10)	H17A—C17—H17D	141.1
C16—C11—S2	120.35 (8)	H17B—C17—H17D	56.3
C12—C11—S2	118.76 (9)	H17C—C17—H17D	56.3
C13—C12—C11	119.02 (11)	C14—C17—H17E	109.5
C12—C13—C14	121.22 (10)	H17A—C17—H17E	56.3
C15—C14—C13	118.82 (11)	H17B—C17—H17E	141.1
C15—C14—C17	120.67 (12)	H17C—C17—H17E	56.3
C13—C14—C17	120.50 (11)	H17D—C17—H17E	109.5
C16—C15—C14	120.77 (11)	C14—C17—H17F	109.5
C15—C16—C11	119.28 (10)	H17A—C17—H17F	56.3
C5—C4—H4	120.7	H17B—C17—H17F	56.3
C3A—C4—H4	120.7	H17C—C17—H17F	141.1
C4—C5—H5	119.5	H17D—C17—H17F	109.5
C6—C5—H5	119.5	H17E—C17—H17F	109.5
C7—C6—H6	119.4		
			
C7A—S1—C2—N3	0.45 (8)	C8—C9—N1—N2	176.74 (9)
C7A—S1—C2—C8	175.95 (9)	C9—N1—N2—S2	−96.08 (10)
C8—C2—N3—C3A	−175.40 (9)	N1—N2—S2—O1	178.74 (7)
S1—C2—N3—C3A	0.03 (11)	N1—N2—S2—O2	−52.67 (8)
C2—N3—C3A—C4	177.18 (10)	N1—N2—S2—C11	62.53 (8)
C2—N3—C3A—C7A	−0.66 (13)	O1—S2—C11—C16	146.33 (9)
N3—C3A—C4—C5	−177.98 (10)	O2—S2—C11—C16	13.11 (10)
C7A—C3A—C4—C5	−0.24 (16)	N2—S2—C11—C16	−101.12 (9)
C3A—C4—C5—C6	−0.91 (17)	O1—S2—C11—C12	−35.40 (10)
C4—C5—C6—C7	1.12 (18)	O2—S2—C11—C12	−168.61 (9)
C5—C6—C7—C7A	−0.15 (17)	N2—S2—C11—C12	77.16 (9)
C6—C7—C7A—C3A	−1.00 (16)	C16—C11—C12—C13	0.85 (17)
C6—C7—C7A—S1	176.79 (9)	S2—C11—C12—C13	−177.42 (9)
N3—C3A—C7A—C7	179.17 (10)	C11—C12—C13—C14	−0.25 (18)
C4—C3A—C7A—C7	1.22 (16)	C12—C13—C14—C15	−1.00 (18)
N3—C3A—C7A—S1	0.98 (11)	C12—C13—C14—C17	−179.74 (12)
C4—C3A—C7A—S1	−176.98 (8)	C13—C14—C15—C16	1.68 (17)
C2—S1—C7A—C7	−178.77 (11)	C17—C14—C15—C16	−179.58 (11)
C2—S1—C7A—C3A	−0.76 (8)	C14—C15—C16—C11	−1.10 (16)
N3—C2—C8—C9	94.63 (11)	C12—C11—C16—C15	−0.18 (16)
S1—C2—C8—C9	−80.57 (10)	S2—C11—C16—C15	178.06 (8)
C2—C8—C9—O3	68.95 (13)	H01—N1—N2—H02	−146.7 (17)
C2—C8—C9—N1	−109.79 (10)	O3—C9—N1—H01	175.5 (13)
O3—C9—N1—N2	−1.96 (16)	H02—N2—S2—O2	179.1 (12)

### Hydrogen-bond geometry (Å, º)

**Table:**

*D*—H···*A*	*D*—H	H···*A*	*D*···*A*	*D*—H···*A*
N1—H01···N3^i^	0.866 (16)	2.013 (16)	2.8717 (13)	171.0 (15)
N2—H02···O3^ii^	0.845 (17)	2.029 (17)	2.8553 (12)	165.7 (16)
C6—H6···O2^iii^	0.95	2.54	3.4142 (15)	154

Symmetry codes: (i) −*x*+1, −*y*, −*z*+1; (ii) −*x*+1, −*y*+1, −*z*+1; (iii) *x*, *y*, *z*+1.
